# Age-, gender- and body site-specific reference values of thermal Quantitative Sensory Testing in the Italian population using the Q-sense device

**DOI:** 10.1007/s10072-023-06929-z

**Published:** 2023-07-14

**Authors:** Giuseppe Cosentino, Elisa Antoniazzi, Laura Bonomi, Camilla Cavigioli, Mariateresa D’Agostino, Massimiliano Todisco, Cristina Tassorelli

**Affiliations:** 1 Department of Brain and Behavioral Sciences, University of Pavia, Pavia, Italy; 2 Translational Neurophysiology Research Unit, IRCCS Mondino Foundation, Via Mondino 2, 27100 Pavia, Italy; 3 Headache Science and Neurorehabilitation Center, IRCCS Mondino Foundation, Pavia, Italy

**Keywords:** Sensory thresholds, Warm detection threshold, Cold detection threshold, Heat pain threshold, Quantitative sensory testing, Age and sex differences

## Abstract

**Background:**

Age-, gender- and body site-specific values of thermal Quantitative Sensory Testing (QST) measures have not yet been reported using the novel and cheap device ‘Q-sense’. Here, we aimed to assess normative values of Q-sense-derived parameters in a representative Italian population.

**Methods:**

QST parameters were measured in 84 healthy participants (42 males; aged 20–76 years) equally distributed into three age groups (18–39, 40–59 and 60–80 years). We explored the Warm and the Cold Detection Thresholds (WDT and CDT, respectively) with the method of limits (MLI) and the method of levels (MLE), and the Heat Pain Threshold (HPT) with the MLI. We tested the trigeminal supraorbital region, the hand thenar, and the foot dorsum on the right body side.

**Results:**

We calculated non-parametric reference limits (2.5–97.5^th^) according to age, gender and tested site. All QST measures were affected by age, gender and tested site. In the extra-trigeminal body sites, females showed lower WDT and higher CDT, while males had higher HPT. Worse sensory discriminative abilities and increased HPT values were found in people aged over 40 on the foot. Age-related differences were more evident with the reaction time–dependent MLI vs. MLE paradigm.

**Conclusions:**

Demographic characteristics must be considered when QST is used in the clinical setting. The definition of reference limits for sensory testing with the Q-sense herein provided can pave the way towards a more widespread use of thermal QST for diagnosing small fiber neuropathy and for identifying patients’ profiles in different chronic pain syndromes.

## Introduction

Thermal Quantitative Sensory Testing (QST) is a psychophysical approach increasingly used to assess the function of small peripheral sensory nerves (i.e., Aδ and C fibers), which mediate the perception of warmth, cold and pain [1–3]. Thermal QST can be applied to determine whether patients show dominant features of thermal sensory deficit or hyperexcitability, thus representing a useful tool in the diagnostic workout of small fiber neuropathies and in the sensory profiling of pain syndromes [4, 5]. More recently, promising QST methods have been developed for evaluating the mechanisms underlying the development and/or maintenance of chronic pain [6]. Normative data so far reported are hardly applicable in the clinical setting, given the differences in methodology and the lack of body site-, age-, gender-, race- and ethnicity-specific values [1, 7–9]. In particular, there are no reference values for the Italian population, there is a paucity of data in elderly subjects, and no age- and gender-specific data are available for several body sites, such as the trigeminal region. It is also noteworthy that site-specific normative data in different age groups and genders have not yet been investigated for QST measures recorded with the more recently commercialized thermo-test device called ‘Q-Sense’. This is an air-based device developed by the Medoc Ltd. Advanced Medical Systems (Israel) to address the need for a less expensive and portable thermo-test device, which can be easily used in the clinical context, also outside of specialized pain centers. A recent study by Pfau et al. assessed the reliability and validity of the Q-Sense by comparing it with the most commonly used water-based Thermal Sensory Analyzer (TSA) device (Medoc Ltd. Advanced Medical Systems, Israel) [10]. Of note, the authors showed an excellent/good agreement between the two devices for both the Warm Detection Threshold (WDT) and the Cold Detection Threshold (CDT) in normal subjects and diabetic patients, and a moderate agreement for the Heat Pain Threshold (HPT) in the patients’ group.

The purpose of this study was to collect reference age- and gender-specific values in a representative sample of the Italian population for three different thermal QST measures (i.e., CDT, WDT, and HPT), using the Q-sense device. Three different body sites were tested: the thenar eminence, the trigeminal supraorbital region, and the foot dorsum. In addition, the two most commonly used QST methods, namely the method of limits (MLI) and the method of levels (MLE), were compared.

## Materials and methods

### Participants

We recruited 84 Italian healthy volunteers (42 men; age range: 20–76 years; mean ± SD: 47.2 ± 18.7 years), divided into three age groups (each of 28 people, with an equal distribution of gender). Age ranges of the three groups were predefined as follows: (i) from 18 to 39 years; (ii) from 40 to 59 years; and (iii) from 60 to 80 years. Based on the medical history and neurological examination (including clinical assessment of tactile, pin-prick and vibratory sensation), exclusion criteria were neurological, psychiatric or systemic diseases, including neuropathies, chronic pain syndromes, migraine, and diabetes. Subjects were also excluded if they were pregnant or breastfeeding, reported bad sleep quality the night before the test, presented any injuries of the skin in the designated test areas, had taken pain perception influencing drugs within the last 72 h, had poor cognition, or were unable to cooperate for the test. Participants were asked to avoid alcohol consumption and intense exercise for 24 h before the test.

### Procedures

Thermal QST was performed following a standardized procedure by two well-trained technicians [7, 8, 11]. The examination was performed in a quiet room with temperature maintained at 22 to 24°C. Before the investigation, participants familiarized with the visual analogue scale (VAS) scoring system and the experimental procedure by having the test demonstrated on their left hand thenar. The test was performed using a 30 × 30 mm air-cooled heat probe connected to a Q-sense Conditioned Pain Modulation (CPM) device (Medoc, Ramat Yishai, Israel). The heat probe was applied on the skin, delivering a constant temperature in the range of 20 to 50°C using a ramp (0.1–2°C/s) and hold strategy. To rule out conditions of abnormally low or high local skin temperature values, these were checked before starting all the experimental procedures by using an infrared thermometer. The skin temperature was in the range between 34 and 37° C in each body site in each tested subject.

Temperature thresholds (i.e., WDT and CDT) and HPT were measured in all participants on the thenar eminence of the right hand, the right supraorbital frontal region, and the dorsum of the right foot, with time intervals of at least 15 min between different body sites. WDT and CDT were quantified first, followed by HPT. To test the WDT and CDT, both the MLI and MLE methods were applied at each body site, in a balanced randomized order between participants. The MLI was used to measure the HPT. All tests were performed in the same session to avoid the influence of day-to-day fluctuations [12]. The thermode was firmly fixed on the different body areas by using an elastic band with velcro. Care was taken to consider body curvatures in placing the probe, such that the best contact between probe and skin surface was achieved. Care was taken in tightening the band the minimum necessary to keep the probe in place without causing a feeling of constriction in the patient. Subjects were informed about how to respond correctly for each testing parameter, and during the test they could not see the change of temperatures on the computer screen.

For the MLI paradigm, the free left hand was used to press a mouse button at the instant in which a specific thermal sensation (warm and cold) was perceived. MLI was performed with increasing stimuli, directed from adaptation range towards sensation range. When assessing the HPT, patients were instructed to press the button as soon as the temperature caused a peak VAS of 50–60 mm. Each temperature test was repeated four times for WDT and CDT and three times for HPT to be certain of a consistent determination of the test temperature. Average values were calculated to obtain single threshold scores. Interstimulus intervals of 8–10 s were kept, and no clues were given to the subject at stimulus onset. For the MLE paradigm, patients were instructed to say ‘yes’ or ‘no’ based on the perception or absence of a specific (warm or cold) stimulus [1, 13]. The interstimulus interval was randomized between 4 and 6 s. The initial temperature step was 3°C. Upon a ‘yes’ patient’s response, the stimulus was repeated with a halved step amplitude. Instead, step amplitude was doubled in case of a ‘no’ response. At every change of direction, step size was halved or doubled at every continuation. This process was halted when amplitude reached a size of 0.1°C, and the MLE threshold was calculated as the mean of the last ‘yes’ and the last ‘no’ response temperatures. A standard sequence of levels stimuli finally results in a single threshold value. In all trials, thermode adaptation temperature was set to 32°C, with rates of temperature change of 1°C/s.

### Statistical analyses

Statistical analysis was performed using the Statistical Package for the Social Sciences software (SPSS, Chicago, IL, USA). Student’s *t* test was adopted to assess possible demographic differences between groups. Since not all QST measures were normally distributed at the Kolmogorov-Smirnov test, reference intervals for the five QST variables (i.e., WDT assessed by MLI and MLE, CDT assessed by MLI and MLE, and HPT) were established using non-parametric reference limits (2.5–97.5^th^) according to age, gender, and body site. Non-parametric tests were carried out to compare QST parameters between different subjects’ groups (age range, gender) and body sites and to assess differences in the values of WDT and CDT by MLI vs. MLE paradigm. The Mann-Whitney test and the Kruskal-Wallis test were used to compare two or more than two groups, respectively. Dunn’s post hoc test for multiple comparisons was used to verify between-subject differences after Kruskal-Wallis test. Spearman’s correlation analyses were carried out to explore the relationships between age and QST measures. For all analyses, *p*<0.05 was considered as significant.

## Results

All participants well tolerated and completed the experimental procedures. Age did not differ between female and male subjects in the three age groups. All subjects in the 18–39 age group were able to perceive the warm and cold stimuli with both MLI and MLE paradigms on all the body sites. In the 40–59 age group, one single female subject did not perceive the cold stimulus applied on the foot with the MLI paradigm. In the 60–80 age group, three male subjects did not perceive the cold stimulus applied on the foot with the MLI paradigm; two of these did not perceive the stimulus even when the MLE paradigm was used. Paradoxical sensations were not reported by any subject. Reference values by age, gender, and body site are shown in Tables 1, 2, and 3.

**Table 1:** Quantitative Sensory Testing measures assessed on the right hand

	Age range	WDT–MLI	CDT–MLI	WDT–MLE	CDT–MLE	HPT
Male	18–39	33.5 ± 0.5 (33.1–35.3)	31.1 ± 0.2 (30.8–31.4)	32.4 ± 0.9 (32.1–34)	31.7±0.2 (30.6–32)	44.1 ± 4.8 (38.7–48.6)
	40–59	34.1 ± 0.8 (33.2–35.8)	30.8 ± 0.5 (29.5–31.4)	32.5 ± 0.5 (32–34)	31.6 ± 0.6 (29.9–32)	43.9 ± 3.6 (39.1 ± 49.2)
	60–80	34.2 ± 1.1 (33.1–35.9)	30.7 ± 0.7 (29.3–31.4)	32.6 ± 1 (32.1–33.4)	31.5 ± 0.6 (29.7–31.7)	44.9 ± 3.5 (37.2–49.1)
Female	18–39	33.4 ± 0.5 (32.9–33.6)	31.2 ± 0.3 (29.6–31.5)	32.3±0.2 (32.1–32.9)	31.7±0.2 (31.3–31.9)	41.1 ± 4.6 (35.5–44.4)
	40–59	33.4 ± 0.6 (32.9–37.5)	30.9 ± 0.4 (28.9–31.3)	32.4 ± 0.4 (32.1–33.5)	31.7 ± 0.6 (30.6–31.9)	43.4 ± 3.2 (38.1–46.8)
	60–80	33.7 ± 0.5 (33–34.4)	31 ± 0.5 (29.6–31.4)	32.7 ± 0.4 (32.1–33.2)	31.7 ± 0.4 (31.3–31.9)	39.5 ± 3.9 (36.2–46.4)

Median ± interquartile range and reference values calculated at 2.5–97.5^th^ percentiles are reported for each variable. Abbreviations: *CDT*, Cold Detection Threshold; *HPT*, Heat Pain Threshold; *MLI*, method of limits; *MLE*, method of levels; *WDT*, Warm Detection Threshold

**Table 2:** Quantitative Sensory Testing measures assessed on the right supraorbital region

	Age range	WDT–MLI	CDT–MLI	WDT–MLE	CDT–MLE	HPT
Male	18–39	33.3 ± 0.4 (32.4–35.4)	31.6 ± 0.2 (31.3–31.9)	32.1 ± 0.2 (32.1–39.8)	31.9 ± 0.0 (31.7–32)	41.8 ± 4.1 (36.6–46.5)
	40–59	34.4 ± 2.1 (32.8–40.4)	31.2 ± 0.4 (30.2–31.8)	32.6 ± 1.3 (32.1–34.8)	31.8 ± 0.2 (31.3–31.9)	41 ± 4.5 (35.5–46.2)
	60–80	34.1 ± 2.9 (32.6–42)	31.3 ± 0.4 (30.4–31.8)	32.3 ± 1.1 (32.1–38.6)	31.7 ± 0.2 (31.1–31.9)	44.3 ± 7.3 (36.2–46.8)
Female	18–39	33.4 ± 1.1 (32.4–37.2)	31.6 ± 0.3 (31.1–31.8)	32.1 ± 0.4 (32.1–34.8)	31.9 ± 0.2 (31.7–31.9)	38 ± 3 (35.5–43)
	40–59	33.4 ± 2.3 (32.9–41.1)	31.4 ± 0.4 (29.5–31.7)	32.3 ± 0.6 (32.1–40.6)	31.9 ± 0.0 (31.5–31.9)	41.1 ± 5.6 (36.3–46.5)
	60–80	33.5 ± 0.9 (32.8–44.2)	31.3 ± 0.4 (30–31.7)	32.5 ± 0.4 (32.1–34)	31.9 ± 0.1 (31.7–32)	39.1 ± 5.1 (35.9–43.7)

Median ± interquartile range and reference values calculated at 2.5–97.5^th^ percentiles are reported for each variable. Abbreviations: *CDT*, Cold Detection Threshold; *HPT*, Heat Pain Threshold; *MLI*, method of limits; *MLE*, method of levels; *WDT*, Warm Detection Threshold

**Table 3:** Quantitative Sensory Testing measures assessed on the right foot dorsum

	Age range	WDT–MLI	CDT–MLI	WDT–MLE	CDT–MLE	HPT
Male	18–39	38.8 ± 5.3 (34.3–43.7)	30.4 ± 1.1 (28.7–31.4)	36.2 ± 5.9 (32.7–41.6)	31.5 ± 0.4 (29.3–31.7)	43.7 ± 2.3 (42.1–47.7)
	40–59	39.7 ± 3.1 (34.9–48.9)	29.3 ± 1.7 (26.1–31.3)	37.5 ± 2.7 (32.5–41.7)	30.4 ± 1.7 (25.1–31.9)	45.1 ± 2.3 (40.4–47.6)
	60–80	42.9 ± 6.8 (35.8–48.9)	30.6 ± 2.3^a^ (22–30.9)	38.4 ± 10.4 (32.3–47)	31.4 ± 1^b^ (24–31.9)	47 ± 4.6 (42.6–49.7)
Female	18–39	34.7 ± 0.4 (34.4–37.2)	31.2 ± 0.4 (28.8–31.5)	32.9 ± 0.9 (32.3–35)	31.7 ± 0.3 (31.5–31.9)	41.6 ± 3.3 (38.3–44)
	40–59	42 ± 6.7 (34.5–45.6)	28.4 ± 2.9^c^ (21.8–31)	39 ± 7.6 (32.7–44.2)	31.3 ± 1.2 (25.1–31.7)	44.9 ± 3 (41.9–48.5)
	60–80	42.1 ± 6.5 (34.7–47.1)	30 ± 2.3 (26.6–31.2)	36.5 ± 7.9 (32.1–45.9)	31.7 ± 0.6 (29.2–32)	46 ± 3.6 (42.4–48.3)

Median ± interquartile range and reference values calculated at 2.5^th^–97.5^th^ percentiles are reported for each variable. Abbreviations: *CDT*, Cold Detection Threshold; *HPT*, Heat Pain Threshold; *MLI*, method of limits; *MLE*, method of levels; *WDT*, Warm Detection Threshold.

^a^Three subjects were excluded as they did not perceive the cold stimulus on the foot

^b^Two subjects were excluded as they did not perceive the cold stimulus on the foot

^c^One subject was excluded as she did not perceive the cold stimulus on the foot

### Age group differences

**Fig. 1:**
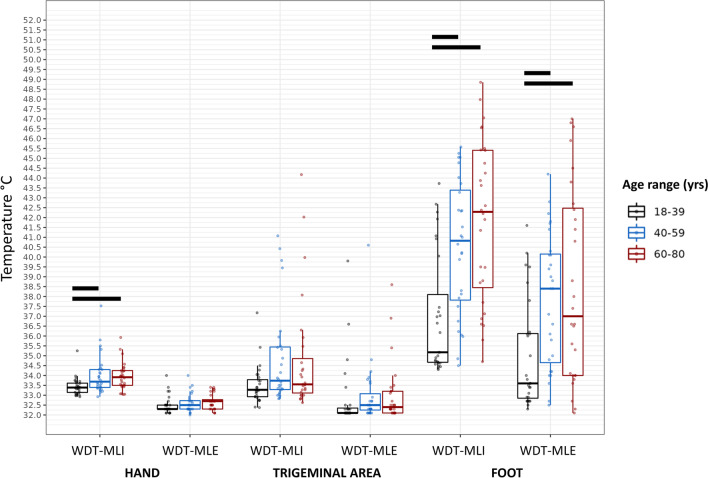
WDT values assessed with the MLI and MLE methods at different body sites. Rectangles represent median, 25th and 75th percentiles, and error bars represent the 2.5th and 97.5th percentiles. Horizontal bars indicate significant differences (see within the text). Abbreviations: *MLI*, method of limits; *MLE*, method of levels; *WDT*, Warm Detection Threshold

**Fig. 2:**
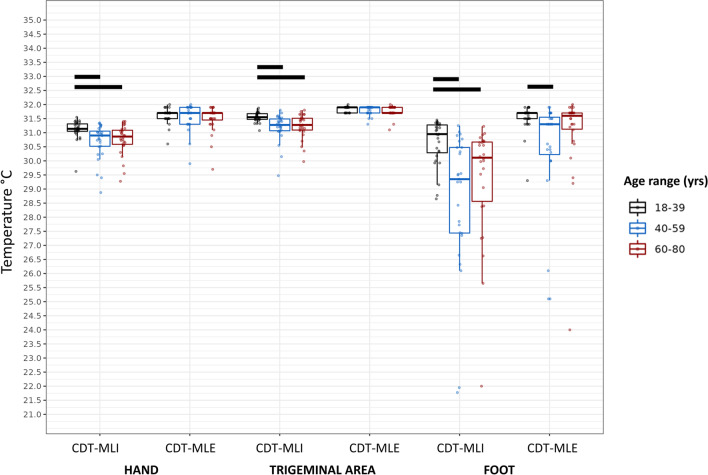
CDT values assessed with the MLI and MLE methods at different body sites. Rectangles represent median, 25th and 75th percentiles, and error bars represent the 2.5th and 97.5th percentiles. Horizontal bars indicate significant differences (see within the text). Abbreviations: *CDT*, Cold Detection Threshold; *MLI*, method of limits; *MLE*, method of levels

When the hand was tested with the MLI, an overall difference among age groups was found with respect to both the WDT (*p*=0.0183) and the CDT (*p*=0.0003) (Figs. 1 and 2). In particular, the 18–39 age group showed lower WDT and higher CDT values as compared to both the 40–59 (*p*=0.0109 for WDT, *p*=0.0023 for CDT) and the 60–80 (*p*=0.0018 for WDT, *p*=0.0045 for CDT) age groups. No significant differences were observed for other QST measures.

When testing the trigeminal region, a difference was detected only with regard to the CDT evaluated by means of the MLI (*p*=.0004). Notably, the 18–39 age group presented higher CDT values as compared to both the 40–59 and the 60–80 age groups (*p*=0.0004 and *p*=0.0027, respectively).

**Fig. 3:**
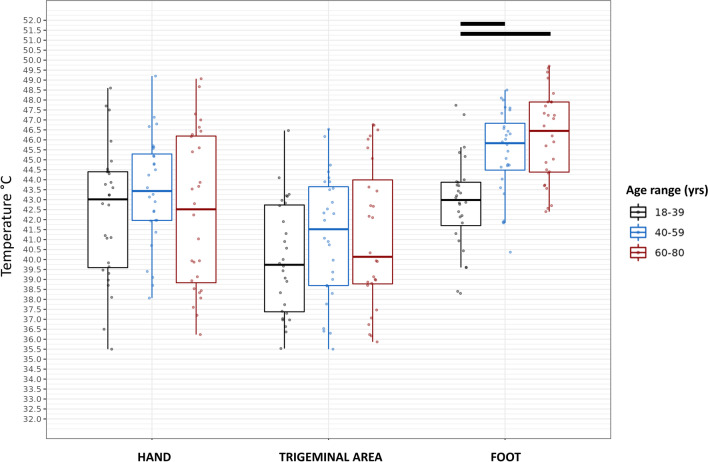
HPT values assessed with the MLI and MLE methods at different body sites. Rectangles represent median, 25th and 75th percentiles, and error bars represent the 2.5th and 97.5th percentiles. Horizontal bars indicate significant differences (see within the text). Abbreviations: *HPT*, Heat Pain Threshold; *MLI*, method of limits; *MLE*, method of levels

When assessing the foot dorsum, an overall difference among groups was observed regarding all QST parameters, particularly WDT and CDT with the MLI approach (*p*=0.0051 and *p*=0.0126, respectively), WDT and CDT with the MLE paradigm (*p*=0.0085 and *p*=0.0398, respectively), and HPT (*p*=0.0002) (Figs. 1, 2, and 3). Except for the CDT with the MLE, post hoc analyses showed that the 18–39 age group had lower WDT and higher CDT values as compared to both the 40–59 (*p*=0.0013 for the WDT-MLI; *p*=0.0001 for the CDT-MLI; *p*=0.0050 for the WDT-MLE; *p*=0.0007 for the HPT) and the 60–80 age groups (*p*=0.0001 for the WDT-MLI; *p*=0.0036 for the CDT-MLI; *p*=0.0039 for the WDT-MLE; *p*=0.0001 for the HPT). In addition, using the MLE paradigm, the 18–39 age group showed higher CDT values as compared to the 40–59 age group (*p*=0.0068); however, it is noteworthy that, as aforementioned, two male subjects in the 60–80 age group were not included in the analysis because they did not perceive the cold stimulus on the foot.

### Gender differences

With respect to the male group, female subjects showed lower WDT values on the hand when assessed with the MLI method (*p*=0.0034) and on the foot when evaluated with the MLE paradigm (*p*=0.0344), thus indicating a better ability to recognize the warm stimulus in the female cohort. As regards the CDT, higher values were recorded on the foot in the female group by means of the MLI paradigm, therefore indicating a better ability to recognize the cold stimulus in this gender (*p*=0.0178). Furthermore, female subjects had lower HPT values on the hand (*p*=0.0002) and on the trigeminal area (*p*=0.005).

### Body site differences

An overall difference among different body sites was reported for all QST measures (*p*<0.0001 for WDT and CDT detected with both the MLI and MLE, and for HPT). Using both the MLI and MLE paradigms, WDT values were higher on the foot compared to both the hand (*p*<0.00001) and the trigeminal area (*p*<0.00001), with no difference between the hand and the trigeminal area.

By means of both the MLI and MLE methods, CDT values were lower on the foot with respect to both the hand (*p*=0.0001 with the MLI and *p*=0.0290 with the MLE) and the trigeminal area (*p*<0.0001 with both paradigms). Moreover, subjects showed lower values on the hand compared to the trigeminal area (*p*<0.0001 with both paradigms).

Also, higher HPT values were shown on the foot compared to both the hand (*p*=0.0004) and the trigeminal area (*p*<0.0001), as well as on the hand with respect to the trigeminal area (*p*=0.0013).

### Differences between MLI and MLE paradigms

Regarding the WDT, lower values were recorded with the MLE paradigm on the hand, trigeminal area and foot (*p*<0.0001 for all body sites). When assessing the CDT, higher values were found with the MLE paradigm (*p*<0.0001 on all body sites).

### Correlation analyses between age and QST measures

Positive correlations between age and WDT-MLI were recorded on the hand (*rho*=0.34, *p*=0.0012), on the trigeminal area (*rho*=0.24, *p*=0.0027), and on the foot (*rho*=0.51, *p*<0.0001). Negative correlation between age and CDT-MLI were observed on all body sites (hand: *rho*=−0.33, *p*=.0023; trigeminal area: *rho*=−0.36, *p*=0.0006; foot: *rho*=-0.39, *p*=0.0002). With regard to the WDT-MLE, positive correlations with age were found on the trigeminal area (*rho*=0.24, *p*=0.0306) and the foot (*rho*=0.36, *p*=0.0006). Finally, the HPT positively correlated with age only on the foot (*rho*=0.51, *p*<0.0001).

## Discussion

This study is the first to assess normative values of thermal QST in the Italian population, and the first to report gender- and age-specific reference values in healthy subjects using the Q-sense device. As a novelty, we also assessed both gender- and age-specific reference values in the trigeminal region, considering that only gender differences have so far been explored at this level [14].

Our findings clearly showed that demographic factors (e.g., age and gender) and the tested body site greatly affected thermal QST measures, thus specific reference values become necessary when thermal QST is used in a clinical setting. The availability of population-specific QST data may also be relevant considering ethnic and socio-cultural differences in pain experience [15, 16], as well as disparities in somatosensory profiles among different ethnic groups [17].

It is well known that thermal QST values change with age [1, 8, 9]. Our results showed that age-related differences were more evident when testing the foot. Indeed, at this level sensory discrimination of both warm and cold stimuli was lower with both the MLI and MLE methods in subjects older than 40 years compared to the younger group. In the other body sites, a reduced discriminative ability of the warm (on the hand) and of the cold (both on the hand and on the trigeminal region) stimulus was observed only using the MLI paradigm in subjects older than 40 years. The difference observed when using the MLI and MLE methods on the hand and trigeminal region can be partially explained by shorter reaction times in younger subjects [1, 18]. Of note, the existence of age-related differences with both the MLI and the MLE methods on the foot may be in line with the hypothesis that a physiological reduction of small nerve fiber density with a length-dependent pattern might occur with advanced age, similarly to what occurs to larger myelinated nerve fibers [19]. This assumption could also underlie the parallel finding of increased HPT values in subjects over the age of 40 and of a positive correlation between age and HPT, exclusively on the foot.

Gender differences in QST measures have already been reported by previous studies [8, 20, 21]. We showed that females had better sensory discriminative abilities than males in the extra-trigeminal body sites, despite differences between the MLI and MLE paradigms. In particular, a better ability to perceive warm stimuli was observed on the hand with the MLI method and on the foot with the MLE paradigm, while for cold stimuli a better sensory discrimination was recorded only on the foot with the MLI method. As compared to the male group, female subjects also showed reduced HPT values both on the hand and on the foot, but not on the trigeminal site, in agreement with the evidence of lower pain thresholds in females [22, 23].

Site differences were observed for warm and cold sensory thresholds with both the MLI and MLE methods, given the reduced sensory discriminative abilities on the foot compared to both the hand and the trigeminal region. A discrepancy between the hand and the trigeminal region was also found for the cold sensory threshold, again with both the MLI and MLE paradigms. Site differences in QST measure by means of the MLI can be partly due to variations in the distance between the tested body site and the brain. Thermal sensation is indeed conveyed via the slowly conducting afferent fibers, so the greater the distance, the greater the impact on reaction time [24]. Nonetheless, differences in QST parameters were also observed with the MLE, suggesting that between-site differences in thermal sensory discrimination actually exist. The finding of lower HPT values on the trigeminal region vs. the hand and on the hand vs. the foot corroborates the idea that body site influences this additional QST measure. However, since the HPT was only assessed using the MLI, variations in the distance between the tested site and the brain could explain these latter body site-related differences, in keeping with Defrin et al. showing that site differences in the HPT values may be observed only when the MLI is applied [24].

Finally, as expected significant lower values for warm and significant higher values for cold sensation thresholds were recorded with the MLE vs. the MLI paradigm on all body sites, in agreement with previous QST findings obtained using the water-based thermal sensory analyzer devices [1, 18].

Some considerations and limitations of the study deserve to be discussed. First, according to Pfau et al. [10], we found that reference values of subjects older than 40 years can exceed the low cut-off value (20°C) of the Q-Sense on the foot. However, and differently from these authors, this finding was found in both males and females in our study, albeit more often in men. Thereby, the sensitivity of the Q-sense to detect cold hypoesthesia in subjects older than 40 years is limited, and this aspect has to be considered for the clinical use. Second, as in the work by Pfau et al. [10], here we tested the HPT only using the MLI method, also to avoid that the application of painful suprathreshold stimuli during the MLE paradigm could more easily induce adaptation phenomena and affect other measures. Moreover, studies using the reaction time-free MLE to assess the HPT are not currently available for the Q-sense device. Finally, though previous studies showed no significant side-to-side differences in QST values in the healthy subjects [25, 26], here we did not test side-to-side differences. Furthermore, despite the high overall number of subjects enrolled, subdivision by gender and age made subgroups sample size relatively small. Therefore, future investigations on larger samples, possibly multi-centre studies on people from different countries, will be needed to confirm our data and establish more robust normative values to be used in the clinical setting. Validation studies in patients with different pathological conditions are also required to assess the sensitivity and specificity of the different QST measures assessed by the Q-sense device.

## Data Availability

The datasets presented in this study will be available in the Zenodo repository.
